# When barriers fail: the role of endothelial dysfunction in rare pediatric neuromuscular diseases

**DOI:** 10.3389/fmed.2026.1834381

**Published:** 2026-07-20

**Authors:** Aleksandra Agafonova, Alessia Cosentino, Chiara Prinzi, Claudia Parano, Angela Trovato Salinaro, Maria Concetta Scuto, Giordana Riccioli, Gabriella Lupo, Carmelina Daniela Anfuso

**Affiliations:** 1 Department of Clinical and Experimental Medicine, University of Catania, Catania, Italy; 2 Department of Biomedical and Biotechnological Sciences, University of Catania, Catania, Italy; 3 Department of General Surgery and Medical-Surgical Specialties, University of Palermo, Palermo, Italy; 4 Department of Medicine and Surgery, Kore University of Enna, Enna, Italy; 5 Department of General Surgery, University Hospital Policlinico G.Rodolico-San Marco, Catania, Italy

**Keywords:** blood-brain barrier, blood-nerve barrier, Duchenne muscular dystrophy, endothelial dysfunction, inflammatory demyelinating polyneuropathy, Kawasaki disease, pediatric neuromuscular diseases, spinal muscular atrophy

## Abstract

Rare pediatric neuromuscular diseases represent a heterogeneous group of disorders in which endothelial dysfunction and blood-tissue barrier alterations can contribute to disease onset and progression. The vascular endothelium, which lines blood vessels, plays a central role in regulating blood flow, immune cell trafficking, and maintaining the integrity of both the blood–brain barrier (BBB) and blood-nerve barrier (BNB). While BBB and the BNB share structural similarities, BNB endothelial cells are more restrictive due to a higher density of junctional complex proteins. Dysfunction of these barriers can lead to inadequate perfusion, neuroinflammation, and increased endothelial permeability. Evidence from rare conditions such as Duchenne muscular dystrophy (DMD), spinal muscular atrophy (SMA), Kawasaki Disease (KD), chronic inflammatory demyelinating polyneuropathy (CIDP), and acute inflammatory demyelinating polyradiculoneuropathy (AIDP) suggests that vascular and barrier abnormalities influence disease severity. However, the precise mechanisms underlying BNB disruption remain poorly understood for several disorders. Understanding these processes not only provides insights into disease pathophysiology but also highlights potential diagnostic and therapeutic targets. This review summarizes current knowledge on endothelial and barrier alterations in rare pediatric neuromuscular diseases, emphasizing the need for further studies to elucidate the involvement of BNB and guide future clinical interventions.

## 1 Introduction

Biological barriers play a crucial role in maintaining homeostasis and protecting tissues from potentially harmful agents. Among these, the blood–brain barrier (BBB) and the blood-nerve barrier (BNB) are highly specialized interfaces that regulate the exchange of molecules between the bloodstream and the nervous system, preserving the integrity of neural environments.

The BBB is a dynamic and highly selective barrier that regulates the molecular exchange between the bloodstream and the central nervous system (CNS), maintaining neural homeostasis and preventing the entry of potentially harmful agents (1). The BBB is composed of endothelial cells connected by specialized tight junctions (TJs) and supported by pericytes and astrocytes, key components of the neurovascular unit (NVU) (2).

In contrast, the BNB is a highly selective structure located at the level of the peripheral nervous system and, although functionally similar to the BBB, presents distinct structural features. It is mainly composed of endothelial cells, pericytes, Schwann cells and basal lamina. The BNB undergoes maturation after birth and differs structurally from the BBB, notably due to the absence of astrocytes in the peripheral nervous system. Despite this difference, the BNB is considered to be structurally highly restrictive, by limiting drug delivery to peripheral nerves, and its breakdown is also linked to the development of peripheral neuropathies (3, 4).

Although the BNB is often discussed as a single entity, its barrier properties differ across its anatomical compartments: the epineurium, perineurium, and endoneurial microvasculature. Among these, the endoneurial compartment is considered the most functionally analogous to the BBB, as it is characterized by non-fenestrated endothelial cells and higher expression of junctional complex proteins. In addition, peripheral nerve pericytes have been reported to express TJ molecules and transporters, and to exhibit high trans-pericyte electrical resistance values (5). Based on these characteristics, some studies have proposed that endoneurial vessels may display barrier properties comparable to, or under certain experimental conditions potentially more restrictive than, those of the BBB (6). However, the literature remains divided regarding the relative restrictiveness of the BBB and BNB, reflecting differences in the anatomical compartments examined, experimental models, and permeability readouts (3, 7).

Neuromuscular diseases (NMDs) are a heterogeneous group of disorders affecting the motor unit, which includes the lower motor neuron (anterior horn), the peripheral nerve, the neuromuscular junction (NMJ), and muscle tissue (8, 9). Pediatric NMDs, such as Duchenne muscular dystrophy (DMD), spinal muscular atrophy (SMA), and hereditary neuropathies (such as Charcot–Marie-Tooth), are conditions classified as rare and characterized by progressive muscle weakness or other motor deficits. Clinical presentation varies depending on the patient’s age, disease subtype and the anatomical region affected. NMDs can manifest prenatally or postnatally, with muscle weakness becoming more evident after the age of two, often detected through signs such as frequent falls and difficulty climbing stairs. As the disease progresses, muscle weakness can lead to progressive orthopedic deformities such as toe walking, pes cavus, or triceps surae contractures. Certain clinical features, such as calf enlargement in DMD, may be highly indicative of specific diagnoses. Given the progressive nature of most NMDs, early diagnosis and regular follow-up are essential, and prompt consultation with a pediatric neurologist is essential to ensure appropriate multidisciplinary management and to improve long-term outcomes (10–12).

In this context, biological barriers play a critical underexplored role in NMD pathophysiology. Although the BBB has been extensively investigated in central neurological disorders, studies addressing CNS-periphery interactions remain comparatively limited. In contrast, there is growing interest in the involvement of BNB in NMDs and its role in peripheral nerve disease progression through alterations in nerve homeostasis and promotion of inflammation and immune cell infiltration (13).

Endothelial dysfunction represents a key mechanism linking barrier impairment to NMD progression. The vascular endothelium, which lines blood vessels, regulates blood flow, immune cells trafficking, and barrier integrity (14). Blood-tissue barriers, particularly the BBB and the BNB, share structural similarities due to the specialization of their endothelial cells. Notably, some studies have reported a high density of intercellular adherens (AJ) junctions and TJ junctions, along with expression of associated proteins such as claudins (claudin 1, 2, 5, and 19), occludin, scaffolding proteins (ZO-1, ZO-2), and junctional adhesion molecules (JAM-A) in BNB endothelial cells (6).

These structural characteristics are essential for maintaining barrier selectivity and controlling leukocyte trafficking. Leukocyte passage across the microvascular endothelium is critical for immune surveillance and the tissue response to injury, inflammation, or infection. However, in pathological states, excessive leukocyte migration is associated with autoimmune neuropathies, leading to nerve inflammation and barrier disruption. Experimental autoimmune neuritis (EAN) studies have demonstrated early increases in BNB permeability associated with inflammatory cell infiltration, while patients with chronic inflammatory demyelinating polyneuropathy (CIDP) show reduced claudin-5 expression and altered ZO-1 localization, supporting the involvement of TJ dysfunction in BNB breakdown (15, 16). These proteins, which are essential for endothelial function and markers of altered vascular permeability, have also been found to be downregulated in experimental nerve injury models, including sciatic nerve constriction in rats, where reduced mRNA levels of occludin and claudin-5 have been documented (15, 17). Although derived from traumatic nerve injury models, these findings provide indirect evidence that TJ disruption may contribute to BNB dysfunction.

**Figure 1:**
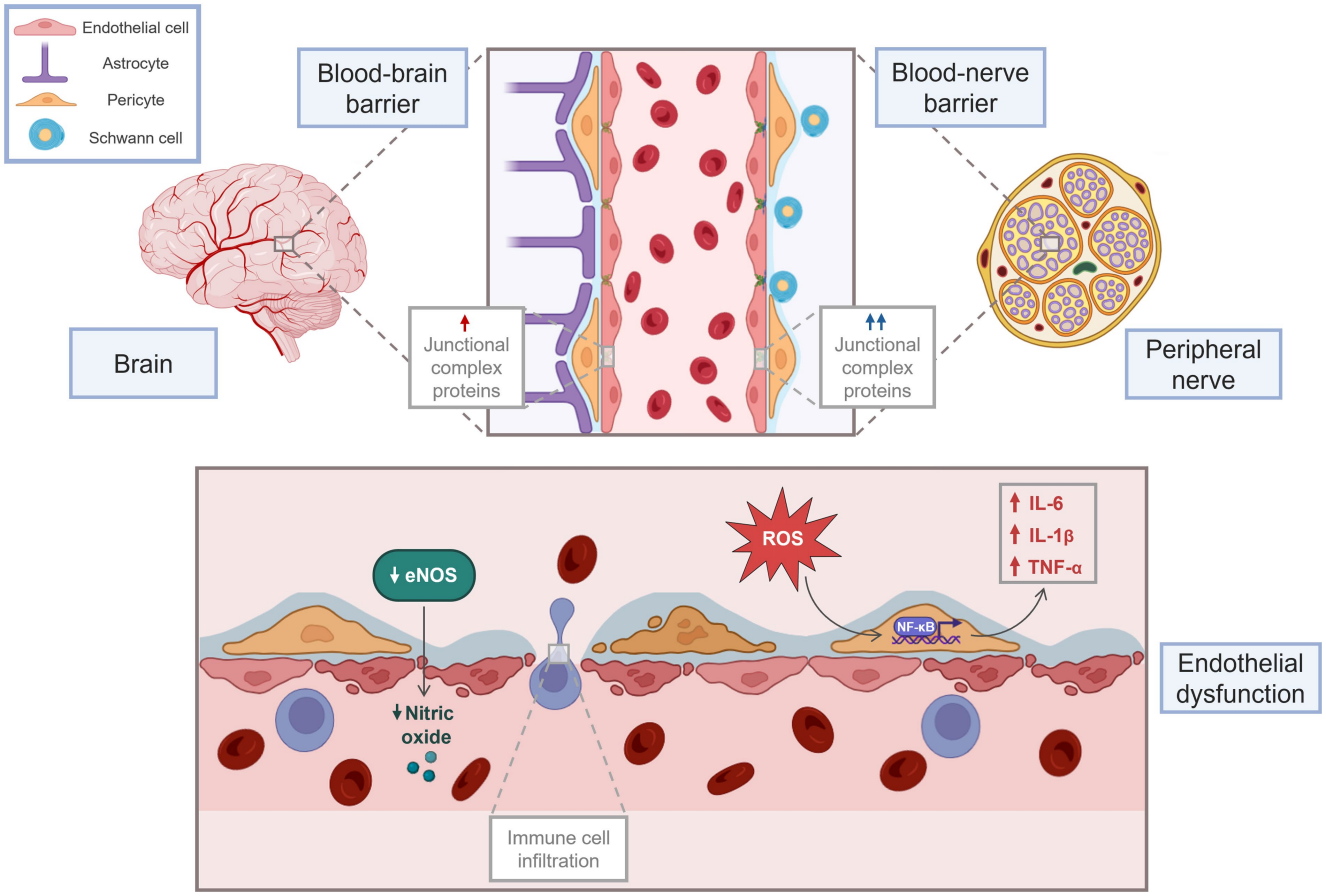
Similarities and differences between BBB and BNB under physiological conditions (upper panel) and signaling pathways activated during endothelial dysfunctions (lower panel). The BBB, composed of endothelial cells, pericytes, and astrocytes, protects the brain by preventing the entry of harmful substances. The BNB is composed of endothelial cells, pericytes, and Schwann cells. Despite the absence of astrocytes, some studies have reported that its endothelial compartment at the endoneurial level exhibits a high density of intercellular AJs, TJs, junction-associated proteins, and JAMs, collectively referred to as junctional complex proteins, contributing to barrier selectivity. NMDs are characterized by endothelial dysfunction, in which junctional complex proteins are reduced, eNOS is downregulated, and inflammation and oxidative stress contribute to endothelial cell disruption. These results in altered vascular permeability, immune cell infiltration, and impaired blood supply. The Figure was generated using BioRender.com.

Peripheral neuroinflammation triggers early changes in the BNB, often leading to the infiltration of various white blood cell types and an increased release of pro-inflammatory mediators such as interleukin 1 (IL-1β), interleukin 6 (IL-6), and tumor necrosis factor (TNF-*α*), which exacerbate nerve injury and partial or total endothelial disruption (18) (Figure 1).

The proper function of endothelial cells in vascular tissues, including the BNB, is largely determined by their ability to regulate the basal release of soluble mediators like nitric oxide (NO). The pulsed generation and release of NO in response to physiological stimuli, such as shear stress or bradykinin, is a key indicator of endothelial cell functionality (19). NO is a major regulator of vascular tone and tissue perfusion and contributes to maintaining vasodilation and blood flow. NO synthase (NOS) exists in three different isoforms: neuronal (nNOS), inducible (iNOS) and endothelial (eNOS), each contributing to NO production in a tissue-specific manner. NO activates guanylate cyclase (GC), by NO-binding to the heme component of GC, leading to cyclic guanosine monophosphate (cGMP) production and downstream regulation of vascular relaxation and microvascular function (20). Alteration in NO signaling has been associated with endothelial dysfunction in several pathological conditions.

In parallel, inflammatory stimuli induce endothelial activation, characterized by increased expression of adhesion molecules and recruitment of immune cells. These cells release pro-inflammatory cytokines and reactive oxygen species (ROS), which activate the nuclear factor kappa B (NF-κB), leading to enhanced transcription of pro-inflammatory genes and increased expression of various adhesion molecules. This cycle of inflammation and oxidative stress can perpetuate endothelial damage and muscle degeneration (21).

Endothelial dysfunction also contributes to hypoxia in muscle tissues due to inadequate blood supply. Normally, hypoxia would stimulate angiogenesis, leading to the formation of new blood vessels (22). However, in DMD this response is impaired, further exacerbating muscle degeneration (23, 24).

Finally, in the pediatric context, the ongoing maturation of the vascular and neurovascular systems, including the NVU and the BBB, may increase vulnerability to endothelial dysfunction compared to adulthood. This can impair tissue growth, neurovascular coupling, and barrier integrity, ultimately disrupting key developmental processes such as myelination, synaptic pruning, and CNS organization, with downstream effects on neuromuscular function and, in severe cases, brain development (25).

## 2 Endothelial dysfunction in rare pediatric neuromuscular disorders

### 2.1 Chronic inflammatory demyelinating polyneuropathy

CIDP is a rare, autoimmune neuropathy comprising a group of heterogeneous disorders with a wide range of clinical phenotypes and variable clinical courses. According to current classification frameworks, CIDP is divided into typical CIDP (t-CIDP) and several clinical variants, including distal acquired demyelinating symmetric (DADS), multifocal acquired demyelinating sensory and motor (MADSAM), focal, motor, and sensory forms (26). It is characterized by motor weakness, sensory alterations and reduced deep tendon reflexes.

Peripheral nerve autoimmune disorders, including CIDP, are strongly associated with increased permeability and functional disruption of BNB. *In vitro* and human biopsy studies indicate that patients with CIDP exhibit a downregulation of TJ proteins, particularly claudin-5, and decreased TEER in peripheral nerve microvascular endothelial cell models (PnMEC), suggesting BNB disruption (27).

Impairment of the BNB leads to immunoglobulins, cytokines, chemokines, and immune cells entering the peripheral nervous system, which is a key event in the pathological process of CIDP (28). Once these circulating factors cross the disrupted barrier, they trigger a cascade of immune-mediated events: macrophages and T/B lymphocytes infiltrate the endoneurium, where they interact with Schwann cells and myelin (29). The recruited immune cells, together with deposited antibodies amplify local inflammation and directly attack myelin sheaths, resulting in segmental demyelination. This sequence of barrier disruption followed by immune cell infiltration represents a central mechanistic link between BNB breakdown and the characteristic neuropathology of CIDP (3, 30).

Standard treatments for CIDP include corticosteroids, intravenous immunoglobulins (IVIg) or subcutaneous immunoglobulins (SCIg), and plasma exchange (PLEX). IVIg is considered a first-line therapy, while corticosteroids, although effective, are less commonly used due to their side effects. Plasma exchange may be an alternative for patients who do not respond to other treatments. Furthermore, rituximab has been shown to be effective in patients who do not respond to conventional treatments (31). However, these treatments primarily act by modulating immune responses and reducing pathogenic autoantibody activity rather than directly restoring or strengthening BNB integrity. Consequently, given the increasing evidence linking BNB dysfunction to CIDP clinical severity, therapeutic strategies specifically targeting BNB function may represent a promising approach, particularly during the early stages of the inflammatory process.

### 2.2 Acute inflammatory demyelinating polyradiculoneuropathy

Acute inflammatory demyelinating polyradiculoneuropathy (AIDP) is a rare, acute immune-mediated demyelinating disorder of the peripheral nervous system and represents the most common Western variant of Guillain-Barré syndrome (GBS). The disease is characterized by a rapid onset, with maximal disease severity typically reached within 4 weeks, distinguishing it from CIDP, which usually progresses beyond 8 weeks. Compared with CIDP, AIDP exhibits a more rapid clinical course and more frequently presents with cranial nerve involvement, particularly bilateral facial weakness (32).

Clinically, AIDP usually presents appendicular and truncal weakness, which may progress to paralysis, accompanied by sensory deficits, respiratory failure, facial paresis, and dysautonomia. In contrast to CIDP, which often follows a chronic relapsing or progressive course, AIDP is generally monophasic and rarely recurs (33).

Neuropathologically, AIDP is characterized by mononuclear leukocyte infiltration into peripheral nerves and nerve roots. Similar to CIDP, monocytes/macrophages constitute the predominant infiltrating cell population in the peripheral nerve, while T- and B- lymphocytes contribute to a lesser extent, leading to macrophage-mediated demyelination. Immune cell entry into peripheral nerves is facilitated by paracellular leukocyte trafficking across a disrupted BNB (3, 34).

The breakdown of BNB facilitates the entry of inflammatory mediators into the peripheral nervous system, promoting segmental demyelination and secondary axonal damage, affecting both motor and sensory fiber (35). Pro-inflammatory cytokines induce the expression of chemokines and cell adhesion molecules at the BNB, including ICAM-1, VCAM-1, E- and P-selectin, thereby enhancing leukocyte-endothelial interactions and immune cell recruitment (36). Elevated levels of TNF-*α*, IL-1β, IL-6, MMP-9, and VEGF have been reported in AIDP and are thought to contribute to barrier dysfunction, myelin injury, and amplification of the inflammatory cascade (37).

### 2.3 Spinal muscular atrophy

SMA is a rare, autosomal recessive disease and the leading inherited cause of infant mortality. SMA manifests with varying severity, classified by the highest motor milestone achieved. SMA type I (infantile SMA) is the most common form, appearing within 6 months of life, with hypotonia, delayed milestones, and feeding difficulties. Infants never sit independently, with a ventilator-free survival median of 8–24 months. SMA type II is characterized by disease onset between 6 and 18 months. Patients can sit but never walk, and most survive into adulthood. SMA type III patients walk at some point during childhood, showing a broad spectrum of severity. SMA Type IV represents the mildest, rarest form, with adult onset, typically after age 30. SMA type 0 is the most severe form, presenting antenatally or neonatally with greatly reduced fetal movements, and is associated with extremely poor survival, typically limited to hours, days or weeks of life (38).

SMA-I is a neuromuscular disease caused by mutations or deletions in the SMN1 gene, which leads to reduced survival motor neuron (SMN) protein levels and motor neuron degeneration. Symptoms typically appear before 6 months of age and often lead to death within 2 years (39).

Emerging evidence suggests that early NVU dysfunction may contribute to disease progression in SMA. The NVU, which is also present in the spinal cord, is a complex multicellular structure composed by endothelial cells, pericytes, astrocytes, neurons, and extracellular matrix components (40). In SMA, SMN protein deficiency and early neuronal dysfunction are hypothesized to disrupt NVU integrity and promote regression of vascular bed. Reduced capillary blood flow can lead to metabolic stress across multiple cellular components of the NVU, compromising astrocyte-mediated regulation of capillary activity. This process may accelerate motor neuron loss in the anterior horn and exacerbate both capillary regression and astroglial dysfunction. Furthermore, sympathetic overactivity contributes to chronic hypoperfusion and ischemia, further damaging motor neurons and astrocytes. Sensory nerve degeneration in SMA-I may also be linked to microvascular dysfunction and NO deficiency, worsening local circulation and increasing neurovascular stress (41).

At the molecular level, SMN deficiency in neurons has been associated with dysregulation of mTOR signaling. This includes reduced axonal mTOR translation via miR-183 and impaired protein synthesis (42). In addition, Thelen et al. found that pyruvate-mediated SMN protein synthesis may occur in an mTOR-dependent manner (43).

Vascular abnormalities appear to play a key role in severe forms of SMA, both in patients and mouse models. Zhou et al. demonstrated that angiogenesis and microvascular defects may arise secondary to SMN deficiency in murine and human endothelial cells (44). Similarly, Allardyce et al. characterized blood-spinal cord barrier (BSCB) in *post-mortem* samples from SMA patients and their findings suggested that vascular defects and associated BSCB impairments are present in the spinal cord of infants with SMA (45). Furthermore, animal studies have reported a significant presence of hypoxic cells in the spinal cord and defects in BSCB, suggesting that vascular impairment contributes to SMA pathogenesis (46).

In line with this, studies in mice show that alterations in the integrity of the BSCB are implicated in various spinal cord pathologies and correlate with motor neuron loss. One study suggests that pericytes play a crucial role in maintaining vascular barrier properties in the spinal cord. However, pericyte coverage and density are reduced in the anterior horn capillaries of the spinal cord in mice, correlating with increased permeability of the BSCB. This permeability is associated with reduced expression of TJ proteins such as ZO-1 and occludin. In Pdgfrb^F7/F7^ mice, which have deficient PDGFRβ signaling, significant reductions in pericyte populations result in a marked increase in BSCB permeability and the accumulation of toxic plasma proteins in motor neurons. These findings suggest a role for pericytes in maintaining the barrier and removing plasma components (47).

Dysfunction of the NMJ has been reported in numerous studies both in mouse models and clinical experience, resulting in weakness and fatigue (48). SMN seems to have a prominent role in NMJ formation and maturation. Therefore, the deletion or mutation of SMN gene leads to NMJ dysfunction, associated to impaired synaptic function and denervation of SMA muscles (49).

The therapeutic strategies for SMA have shifted with the advent of gene-targeted therapy. Traditional therapies focused on supportive approaches, including physical therapy and other forms of rehabilitation to alleviate symptoms (50). In addition, respiratory and orthopedic management were also central, focusing on supporting pulmonary function and correcting skeletal deformities, which are common severe complications in untreated patients (51).

Advances in molecular biology have enabled the development of therapies that enhance the production of SMN protein. A deeper understanding of the genetic basis of SMA has led to the approval of three disease-modifying therapies: Nusinersen, Risdiplam, and Onasemnogene Abeparvovec-xioi. Nusinersen, an intrathecally administered antisense oligonucleotide, and Risdiplam, an oral molecule, both act by correcting the splicing of the SMN2 gene, thereby enhancing the production of SMN protein. Onasemnogene Abeparvovec-xioi, an adeno-associated (AAV)-based gene therapy, delivers a functional copy of SMN1 gene to target cells and is particularly effective when administered in the presymptomatic phase (52, 53).

### 2.4 Duchenne muscular dystrophy

DMD is a rare X-linked neuromuscular disorder caused by mutations in the gene encoding dystrophin, with an incidence of approximately 1 in 3600 young males (54). Dystrophin is a critical protein for maintaining the integrity of muscle fibers (55). Therefore, DMD leads to progressive muscle wasting, determining the loss of ambulation during early adolescence, cardiomyopathy and respiratory failure (56, 57).

DMD remains an incurable disease, despite significant advancement in genetic and molecular research, due to the complexity of the DMD gene and the challenging therapeutic approaches. Current treatments, including corticosteroids, are mainly focused on alleviating the symptoms, but do not treat the root cause of the disease (58).

Endothelial dysfunction has been increasingly recognized as a key pathological feature in DMD patients, even in the early stages of the disease. In children with DMD, the endothelial cells’ ability to regulate vascular tone and maintain vascular health is compromised, leading to a cascade of cardiovascular complications. In fact, the absence of functional dystrophin not only disrupts the structural integrity of muscles, but also impairs vascular function (59). Dystrophin plays a main role in the expression of structural and signaling proteins associated with the dystrophin-glycoprotein complex, concerning NO, ROS and Ca^2+^ signaling pathways (54).

NO is a critical molecule for vasodilation and skeletal muscle physiology. Normal muscle’s excitation-contraction coupling is modulated by NO, which also maintains mitochondrial function, controls glucose uptake in muscles and vascular perfusion during contraction (60). Skeletal muscle exhibits low amounts of eNOS, mainly associated with the vascular endothelium, and iNOS levels; however, inflammatory cells that contain iNOS infiltrate the dystrophic muscle, resulting in significantly increased amounts of this isoform in the skeletal muscle tissue (61). In contrast, nNOSμ represents the predominant splicing form expressed in skeletal muscle and is associated with the sarcolemma. nNOS interacts with *α*-syntrophin and dystrophin, and in DMD the loss of these two proteins causes a mislocalization of nNOS from the sarcolemma to the cytosol and a down-regulation of its expression, leading to a reduction of NO production (57, 62). The dysfunction of the nNOSμ-cGMP-PDE5 axis contributes to reduced vasodilation and poor muscle perfusion, further exacerbating muscle damage (55).

Several studies have demonstrated that the lack of dystrophin in mdx mice, a murine model of DMD caused by a point mutation in the DMD gene, leads to vascular dysfunction. In mdx brain, ultrastructural alterations of the vessel wall have been described, including endothelial abnormalities, dissociation of the TJs, and an increase in vesicular and vacuolar transcellular transport (63). Vascular abnormalities occur as a result of decreased vascular density, decreased expression of eNOS and nNOS, and impaired NO-mediated vasodilation, and reduced blood flow (64, 65). The migration of eNOS to caveolae is essential for its proper activation and the regulation of vascular function. Caveolin-1 plays a central role in this process by modulating the interaction with eNOS (66). While dystrophin is not directly involved in the migration of eNOS to caveolae, disruptions in dystrophin expression, particularly in endothelial cells, can impair eNOS function and contribute to vascular dysfunction. Therefore, dystrophin may indirectly support the proper localization and function of eNOS in the endothelium.

Oxidative stress and inflammation are central mediators of endothelial damage in DMD. Oxidative stress, characterized by an imbalance between reactive species production and antioxidant defenses, is a hallmark of DMD. Compared to normal muscles, muscles from animal models and DMD patients generate noticeably higher levels of free radicals. In the mdx model it has been seen that the absence of dystrophin and the mislocalization of nNOS affect microtubule organization, leading to NADPH oxidase 2 (NOX2) increased activity with higher ROS production (67, 68). In particular, mdx cardiac muscle and mdx skeletal muscle fibers exhibit increased NOX activity as a source of oxidative stress (69–71). The absence of dystrophin and delocalization of nNOSμ reduce NO production in muscle cells, impairing vasodilation during exercise and leading to ischemic damage. Compensatory overproduction of NO by iNOS in inflammatory cells contributes to pathological levels of RNS, inducing nitrosative stress (72).

TNF-*α* plays a crucial role in inducing iNOS expression in neutrophils, macrophages, and other cell types, activating the transcription factor NF-κB. NF-κB, increasing the expression of iNOS and other pro-inflammatory mediators, drives macrophages toward the M1 phenotype. iNOS metabolizes arginine into citrulline and free-radical NO and the high amounts of NO produced by iNOS can significantly alter the redox environment in injured tissue and react with other free radicals, amplifying their reactivity and toxicity, inducing muscle membrane lysis (11, 73).

Cumulative oxidative damage disrupts endothelial cell TJs, promoting vascular leakage. Immune cell infiltration, particularly by macrophages and neutrophils, perpetuates a pro-inflammatory microenvironment. Oxidative stress and elevated levels of pro-inflammatory cytokines such as TNF-*α*, IL-1β and IL-6 induce endothelial activation, characterized by upregulation of adhesion molecules (e.g., ICAM-1) that exacerbate leukocyte adhesion and extravasation (74). The pro-inflammatory and pro-oxidative environment also generates a pro-thrombotic state by impairing endothelial anticoagulant mechanisms and promoting platelet activation, aggregation, and fibrin deposition. These processes collectively contribute to microvascular occlusions, reduced tissue perfusion, and exacerbated ischemic injury in DMD (75).

DMD muscle models exhibit higher cytosolic calcium concentration, which induces mitochondrial dysfunction and triggers degradative pathways, including calcium-dependent calpain protease and phospholipase A_2_ (PLA_2_) and mitochondrial-dependent necrosis (58, 76). Dystrophic muscle displays an increased PLA_2_ activity and it seems to be related to sarcolemmal lipid disruption and increased permeability, and it is possible that it can increase ROS production in muscle (77). The absence of dystrophin makes vascular smooth muscle cells more susceptible to hypoxia-induced Ca2+ (78). Impaired angiogenesis has been observed in DMD, including lower vascular density and impaired expression of pro-angiogenic factors like vascular endothelial growth factor (VEGF) and Hypoxia-Inducible Factor 1 *α* (HIF-1α) (63). These deficiencies exacerbate the ischemic environment in muscles, which impairs regeneration and exacerbates fibrosis (79). Enhancing angiogenesis through VEGF therapies and other pro-angiogenic strategies is being investigated as a potential treatment to increase blood flow (80).

In DMD the absence of dystrophin affects not only muscle fibers but also vascular barriers and synaptic integrity, contributing to disease progression and multisystem involvement. Early studies demonstrated that dystrophin is essential for the maturation of synapse and neurotransmission at the neuromuscular junction (NMJ), and that NMJ alterations are correlated with impaired muscle contraction force in DMD (81, 82). In addition, vascular dysfunctions in the brain, in particular BBB alterations, and pathological angiogenesis have been reported in mdx mouse model (83). Studies have shown that reduced expression of dystrophin-associated proteins and a deficiency of the dystrophin isoform Dp71 at glial endfeet contribute to BBB dysfunction (84, 85). While vascular dysfunction and endothelial alteration have been described in DMD, there is currently no direct evidence demonstrating pervasive BNB disruption in DMD models or patients. More research is needed to determine whether peripheral nerve barriers are affected in dystrophinopathies.

### 2.5 Pediatric vasculitis and Kawasaki disease

Systemic vasculitis refers to different diseases characterized by inflammation of blood vessels, resulting in neuromuscular complications. Kawasaki disease (KD), a rare mucosa-cutaneous lymph node syndrome, is a particular form of vasculitis and represents the most common coronary artery disease in children, with a highest prevalence among children aged under 5 years and in Asia (86).

While KD and other pediatric vasculitides are not NMDs per se, they can result in neuromuscular complications due to inflammation in blood vessels. In fact, the hallmark of KD is widespread inflammation of medium-sized arteries, particularly coronary arteries. The precise etiology remains unclear; however, genetic predisposition and environmental factors are found to be involved.

Endothelial dysfunction is a critical component of KD pathogenesis and a predictor of long-term vascular outcomes. Endothelial cells are targets of immune-mediated injury, resulting in pro-thrombotic states. KD-induced inflammatory factors (e.g., IL-1, IL-6, TNF-*α*, IFN-*γ*) accumulate in coronary artery endothelial cells, which lead to production of ROS and consequently promote vascular endothelial cells dysfunction (87–90). Many matrix metalloproteinases (MMPs) are highly expressed in KD and damage components of basal membranes and extracellular matrix proteins, disrupting the vascular wall. In particular, IL-1, IL-6 and TNF-α stimulate endothelial cells to produce MMP-9 (91).

Pro-inflammatory cytokines also stimulate the endothelial cells to upregulate adhesion molecules (E-selectin, ICAM and VCAM-1). Their over-expression enhances the recruitment of neutrophils and monocytes, amplifying vascular inflammation (92). The levels of E-selectin, ICAM-1, and VCAM-1 in serum or plasma correlate with the severity of endothelial dysfunction in KD, making them potential biomarkers for diagnosing active disease.

In the acute stage of Kawasaki disease, monocytes and macrophages are the main cells involved in arterial inflammatory infiltration. These cells activate NOX, leading to ROS production. NOX causes an activation of arachidonate cascade, producing reactive oxygen metabolites (ROMs), including hydroxyperoxide, a marker of oxidative damage (93). Infiltrating inflammatory cells also express iNOS, producing unstable NO, which leads to peroxynitrite production (94). Peroxynitrite exacerbates vascular dysfunction, damaging lipids and proteins and uncoupling eNOS (95).

Oxidative stress activates IκB kinase (IKK) complex, which phosphorylates IκB, leading to its ubiquitination and degradation via the proteasome. This degradation releases NF-κB, which translocates into the nucleus of inflammatory cells. Once in the nucleus, NF-κB binds to specific DNA sequences in the promoters of target genes, initiating the transcription of pro-inflammatory cytokines, adhesion molecules and other mediators of inflammation (96).

Serum levels of high mobility group box 1 (HMGB1) and expression levels of gene encoding receptor for advanced glycation endproducts (RAGE) are significantly high in children in KD (97). Furthermore, RAGE activation is associated with NF-κB stimulation.

While endothelial involvement is well established in KD, the present review did not provide evidence for a direct impairment of the BNB, indicating that further investigation is needed to determine whether peripheral neurovascular interfaces are affected.

## 3 The role of endothelium in BNB dysfunction

### 3.1 Alterations in endothelial permeability and changes in tight junction, inflammatory response and oxidative stress

As previously mentioned, BNB plays a crucial role in maintaining the homeostasis of the endoneurial microenvironment and protecting peripheral nerves from pathogens and harmful molecules present in the blood. When the BNB is compromised, it can lead to the progression of various neuropathies, particularly inflammatory ones. Studies in animal models and clinical data suggest that BNB impairment is prevalent in inflammatory neuropathies.

For instance, research involving models such as experimental allergic neuritis (EAN) in rats and rabbits has revealed that BNB impairment is among the initial pathological events (98). In these models, injecting myelin antigens (such as P0 or P2 proteins) or gangliosides triggers inflammation of the peripheral nerves. Peripheral neuroinflammation induces early alterations of the BNB, including reduced expression and altered localization of TJ proteins, as well as increased vascular permeability (17). These changes typically precede the infiltration of immune cells, such as lymphocytes and macrophages, into the nerve, a critical step in the development of segmental demyelination and axonal damage. Studies on specific axonal GBS models have shown that anti-ganglioside antibodies formed following infections such as *Campylobacter jejuni* can directly attack peripheral nerve axons once the BNB is damaged (99, 100).

Histopathological examination of nerve biopsies has demonstrated BNB disruption, increased interstitial edema, and infiltration of inflammatory cells, including lymphocytes and macrophages. This breakdown of BNB facilitates the entry of immune mediators into the endoneurial space, promoting immune-mediated damage to myelin and, in some cases, to axons (30). As a consequence, nerve impulse conduction becomes impaired or blocked, leading to clinical manifestations such as muscle weakness, loss of sensation and paralysis. Persistent barrier dysfunction may allow a continuous and unregulated immune attack, which can lead to rapid clinical deterioration and poorer outcomes, as observed in animal models and in patients with GBS and CIDP (101).

Another study showed that patients with the three subtypes of CIDP have damaged microvascular endothelial cells in their peripheral nerves. In particular, there was a reduction in claudin-5 protein levels and TEER values, which measure integrity. This effect was more pronounced in patients with typical CIDP than in those with the other two types (MADSAM and DADS) (28). The BNB consists of TJ complexes between endothelial cells, and their expression is significantly reduced in inflammatory conditions. *In vitro* studies using cellular models have demonstrated the disorganization and reduced density of junction proteins such as claudin-5, occludin and ZO-1 (3). Endothelial dysfunction leads to various pathological states and is associated with increased expression of pro-inflammatory factors, increased endothelial permeability, and increased leukocyte adhesion and monocyte migration (102).

Chen et al. explored the role of the TJ protein Claudin-12 in maintaining barrier integrity and how its deficiency affects TJ function. In particular, reduction in Claudin-12 expression was observed in CIDP patients. Claudin-12 deficiency resulted in a selective loss of other TJ proteins in the myelin barrier. Claudin-19 and PMP22 were decreased in KO mouse models, and TNF-*α* levels were also increased. These findings suggest that a compromised barrier facilitates the entry of cytokines or inflammatory mediators into nerves, promoting sensitization and neuropathy (103).

Several studies have also highlighted a relative involvement of inflammation and oxidative stress in neurodegenerative processes. In this context, a study suggests that genetic variations in genes associated with oxidative stress, inflammation, and neurodevelopment may alter the course of SMA, leading to motor and respiratory problems, and provides further links between these factors and neurodegeneration (104). Oxidative stress also plays a crucial role in DMD. In fact, the accumulation of free radicals leads to the onset of atrophy and weakness (105). It has been shown that circulating markers of oxidative stress such as NO, MDA and 8-isoprostane could be useful markers to assess the progression of DMD. This is because an increase in oxidative stress and a decrease in antioxidant capacity have been observed in patients with DMD (106). In SMA, oxidative stress can lead to impaired mitochondrial function in spinal motor neurons. One study observed the crucial role of mitochondrial defects in the pathogenesis of SMA (107).

### 3.2 Diagnosis of BNB dysfunction and therapeutic implications

Little information is available in the literature regarding BNB dysfunction in pediatric age; however, substantial evidence from adult models and cellular studies provides strong hypotheses that deserve investigation in pediatric NMD context. Recent researches has focused on identifying molecular targets for disease-specific therapies in peripheral neuropathies and chronic neuropathic pain, taking into account the unique biological features of adult human BNB (3). These findings could be also relevant in pediatric populations, particularly in conditions characterized by inflammatory mechanisms, such as CIDP, as well as in metabolic or rare neuropathies, which may involve oxidative stress, glycation, or alterations in the vascular microenvironment.

Such mechanisms imply dysfunction at the BNB level. To assess BNB impairment, *in vitro* studies can be performed using immortalized cell lines. For example, one study evaluated BNB impairment in diabetic peripheral neuropathy (DPN). The authors used thermosensitive immortalized cell lines of vascular endothelial cells and pericytes derived from human sciatic nerves to assess BNB breakdown primarily in response to advanced glycation end products associated with DPN (108).

In recent years, imaging techniques enabling indirect assessment of BNB permeability have also been introduced. Bäumer et al. used dynamic contrast-enhanced perfusion imaging of peripheral nerves to measure blood-nerve permeability (K) and nerve blood volume (NBV) in peripheral neuropathies compared to healthy controls. The findings suggest that this approach may represent a promising tool for future research in peripheral neuropathies (109).

Interventions aimed at stabilizing the BNB may be of considerable interest. For instance, Patritti-Cram et al. found clear evidence that BNB integrity is regulated by stem cells in a non-cell-autonomous manner. Nf1 mutant stem cells, known to increase the production and release of growth factors, impacted surrounding perineural and endothelial cells, leading to BNB breakdown (110).

## 4 Conclusion

In the context of rare pediatric NMDs, the failure of endothelial barriers, particularly the BNB, emerges as a critical link between systemic inflammation, immune-cell trafficking, and nerve damage. BNB dysfunction represents a relevant pathological mechanism in several rare neuropathies, including genetic, inflammatory, and metabolic forms. Although the pediatric literature on BNB is limited and often relies on small-sample studies and animal models that only partially recapitulate human disease, evidence from adult-onset neuropathies and cellular experiments provides strong hypotheses that deserve investigation in pediatric neuromuscular settings.

It is crucial to promote well-designed pediatric studies that integrate multiple complementary approaches, including standardized biomarkers, *in vitro* models derived from juvenile tissue, and less invasive imaging methods to assess BNB permeability in children with neuropathies. Longitudinal studies are needed to clarify whether BNB alteration is a primary driver or a secondary consequence of neuroinflammation and to evaluate its value as a prognostic or therapeutic-response marker.

Developing therapies that selectively target and stabilize the BNB may offer novel therapeutic prospects in these rare conditions. Future work should focus on identifying disease-specific BNB vulnerabilities, optimizing delivery systems for neuroprotective agents across the barrier, and translating preclinical findings into early-phase clinical trials in pediatric populations, with particular attention to age-related differences in barrier biology and immune responses.
